# First Direct Evidence of Accelerated Molecular Aging in Intracranial Aneurysmal Tissue

**DOI:** 10.1111/acel.70231

**Published:** 2025-09-30

**Authors:** Dilaware Khan, Xuanchen Li, Michael Hewera, Sajjad Muhammad

**Affiliations:** ^1^ Department of Neurosurgery Medical Faculty and University Hospital Düsseldorf, Heinrich‐Heine‐University Düsseldorf Düsseldorf Germany; ^2^ Department of Neurosurgery University of Helsinki and Helsinki University Hospital Helsinki Finland

**Keywords:** molecular aging, mTOR, NF‐κB, oxidative stress‐induced DNA damage, telomere length

## Abstract

The risk for cardiovascular diseases increases with age. Various markers for vascular aging have been suggested. However, these markers are not a direct measure of aging in vessels. Telomere length quantification can directly measure vascular aging—the current study aimed to investigate aging in aneurysm tissue by quantifying telomere length. Non‐diseased control vessels and ruptured and unruptured intracranial aneurysm vessels were resected during surgery. Telomere length quantification revealed a shorter telomere length in intracranial aneurysm tissue than in the non‐diseased control vessel. The difference in telomere length between non‐diseased control vessels and intracranial aneurysm tissue remained significant after normalizing for age. Moreover, the intracranial aneurysm tissue showed a lower expression of the aging marker Lamin B1 and a higher expression of the senescence marker P21. Additionally, intracranial aneurysm tissue presented higher activation of mTOR and NF‐κB pathways, which are known to contribute to inflammation and aging. Oxidative stress‐induced DNA damage appeared higher in intracranial aneurysm tissue than in non‐diseased control vessels. Our human data clearly showed increased molecular aging, elevated oxidative stress, and the activation of aging and inflammation‐associated pathways NF‐κB and mTOR in intracranial aneurysm tissue compared to non‐diseased control vessels.

## Graphical abstract

This study provides the first direct evidence of accelerated molecular aging in human intracranial aneurysm tissue, marked by telomere shortening, oxidative DNA damage, and activation of mTOR and NF‐κB pathways. These findings reveal critical aging‐related mechanisms potentially driving aneurysm formation and rupture.

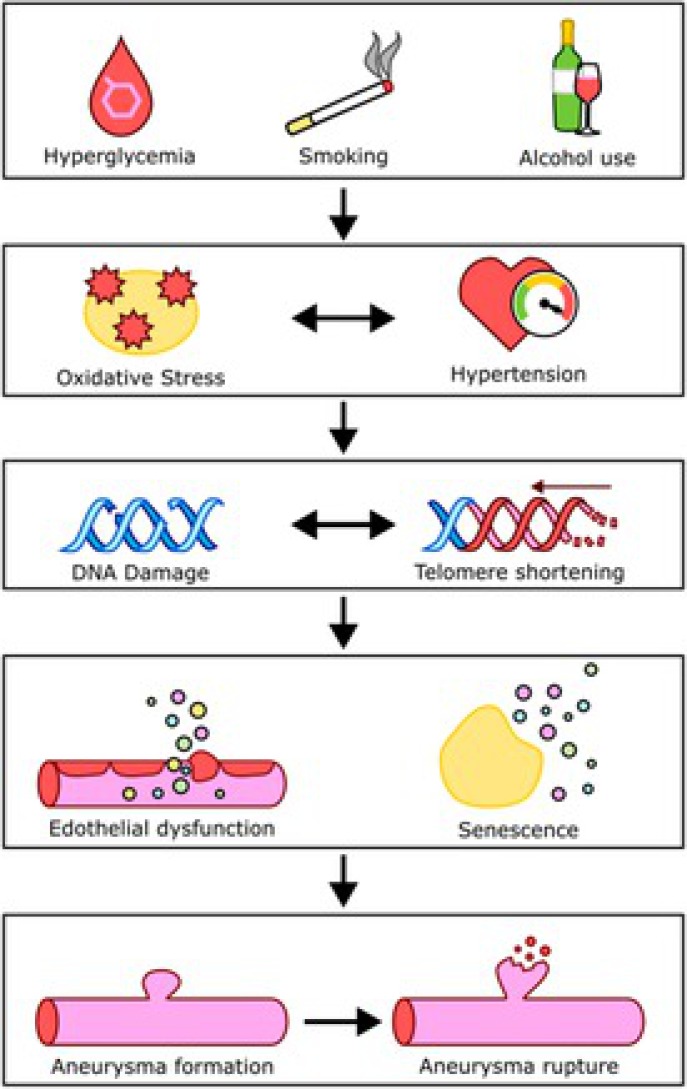

Aging is a significant risk factor for cardiovascular diseases. It is a heterogeneous process affected by many factors, including genetic background, epigenetics, environment, and lifestyle. Vascular aging is gaining academic and clinical interest. However, there is no consensus on determining vascular aging physiologically versus pathologically. Factors such as pulse wave velocity, pulse pressure, arterial augmentation index, carotid intima‐media thickness, carotid plaque, coronary artery calcification, and endothelial dysfunction have been suggested as markers of vascular aging (Climie et al. 2023). However, these are the symptoms “and” or “or” consequences of vascular aging and are not direct evidence of vascular aging.

Quantifying telomere length can determine molecular aging in vessels. Telomeres cap the extremes of chromosomes, providing stability to the DNA and preventing DNA damage (Turner et al. 2019). In healthy conditions, telomeres shorten with each cell division because of the incomplete copying of lagging DNA strands during DNA replication (Turner et al. 2019). When telomeres reach the minimum physiological range, cells stop dividing and enter either senescence or apoptosis (Turner et al. 2019). Pathological conditions such as inflammation and parasitic infection can accelerate telomere attrition (Liu et al. 2019; Asghar et al. 2018). Moreover, cardiovascular risk factors like smoking, alcohol abuse, obesity, and metabolic diseases promote oxidative stress and have been linked to enhanced telomere shortening and endothelial cell senescence (Turner et al. 2019; Khan et al. 2024; Li et al. 2022; Astuti et al. 2017; Clemente et al. 2019). Also, oxidative stress can cause DNA damage and expedite telomere shortening (Zhou, Khan, et al. 2023; Coluzzi et al. 2014). With age, oxidative stress increases in the vasculature due to the imbalance between the oxidant and antioxidant systems. Oxidative stress induces inflammation and activates inflammatory and aging pathways such as NF‐κB and mTOR (Zhou, Khan, et al. 2023; Garcia‐Garcia et al. 2021). This causes chronic low‐grade inflammation in the vascular tissue, termed inflammaging, a hallmark of age‐related cerebrovascular and cardiovascular diseases such as hypertension, atherosclerosis, coronary artery disease, aneurysm, and stroke (Ajoolabady et al. 2023; Barcena et al. 2022).

In the current study, we investigated molecular aging in intracranial unruptured and ruptured aneurysm tissue compared to non‐diseased control vessels resected during surgical procedures from patients. Our study provides the first direct evidence of accelerated molecular aging in vessels of intracranial aneurysms. Moreover, it shows augmented oxidative stress and enhanced activation of aging and inflammation‐associated pathways mTOR and NF‐κB in the intracranial aneurysm tissue.

## 1 Results

**FIGURE 1:**
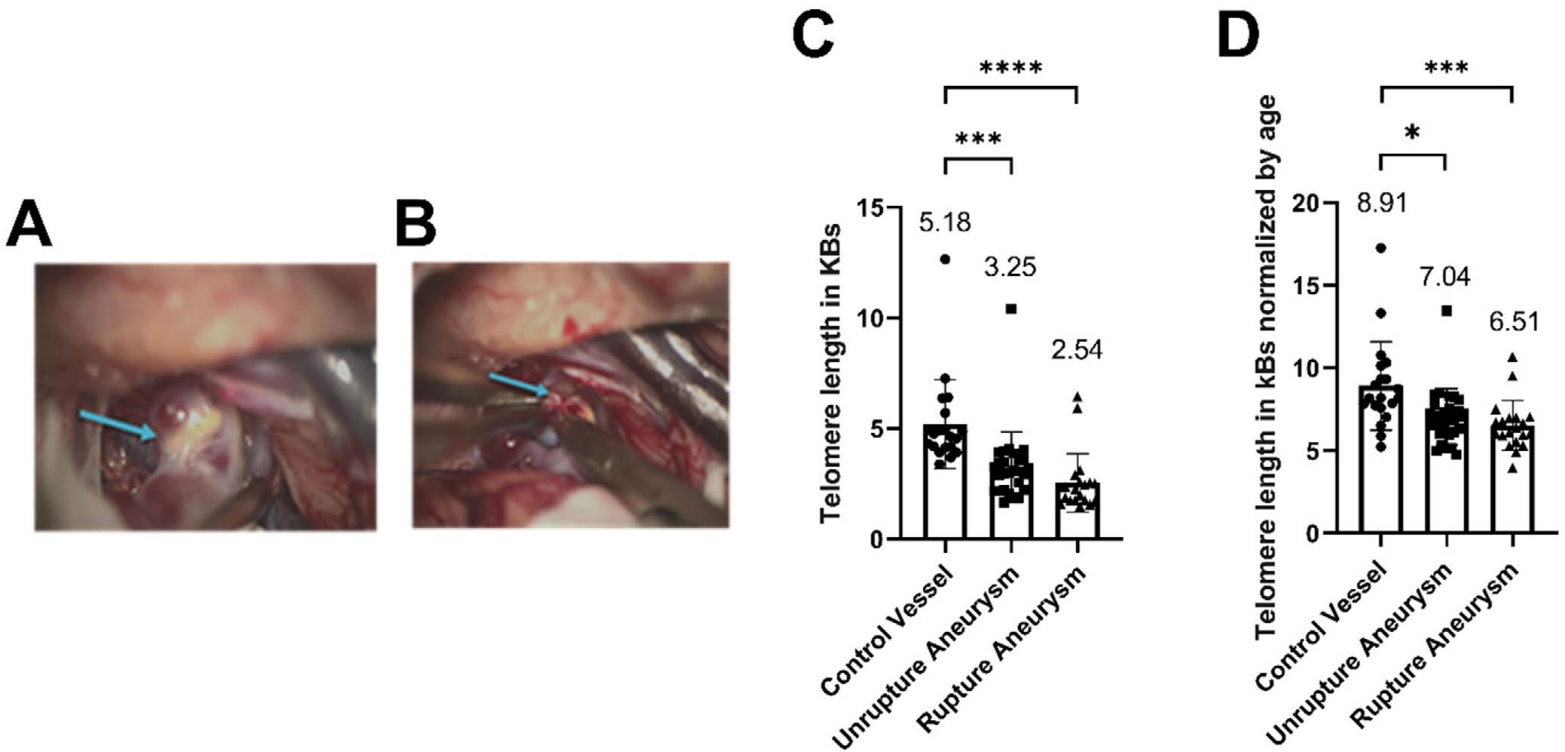
Enhanced molecular aging in intracranial aneurysm tissue. The tissue samples were resected from (A) stable aneurysm area and (B) at the site of rupture. (C) Telomere length in non‐diseased control vessel (*n* = 20), unruptured (*n* = 27), and ruptured (*n* = 20) intracranial aneurysm tissue. (D) Telomere length is normalized by age in the non‐diseased control vessel and unruptured and ruptured intracranial aneurysm tissue. Statistical analysis was performed using the Kruskal–Wallis test with Dunn's multiple comparisons test (*p < 0.05, ***p < 0.001, ****p < 0.0001).

Group demographics are summarized in Table 1. Telomere length was quantified to investigate molecular aging in tissue resected from the stable aneurysm area (Figure 1A) and the site of aneurysm rupture (Figure 1B). The non‐diseased vessels from arteria meningea media were used as control. Telomere length quantification revealed accelerated molecular aging in intracranial unruptured (*n* = 27) and ruptured (*n* = 20) aneurysm tissue compared to the non‐diseased control vessel (*n* = 20) (Telomere length in KBs: Control = 5.18 ± 2.01, unruptured aneurysm = 3.25 ± 1.60, ruptured aneurysm = 2.54 ± 1.33, ***p < 0.001, ****p < 0.0001, Figure 1). Telomere attrition remained significantly higher in intracranial aneurysm tissue after normalization by age (Telomere length normalized by age: Control = 8.91 ± 2.67, unruptured aneurysm = 7.04 ± 1.69, ruptured aneurysm = 6.51 ± 1.50, *p < 0.05, ***p < 0.001, Figure 1B).

**TABLE 1:** Group demographic.

Group	*n*	Mean age ± SD	Male	Female
Control	20	53.60 ± 22.18	5	15
Unruptured Aneurysm	27	54.22 ± 13.48	8	19
Ruptured Aneurysm	20	56.75 ± 10.67	3	17

**FIGURE 2:**
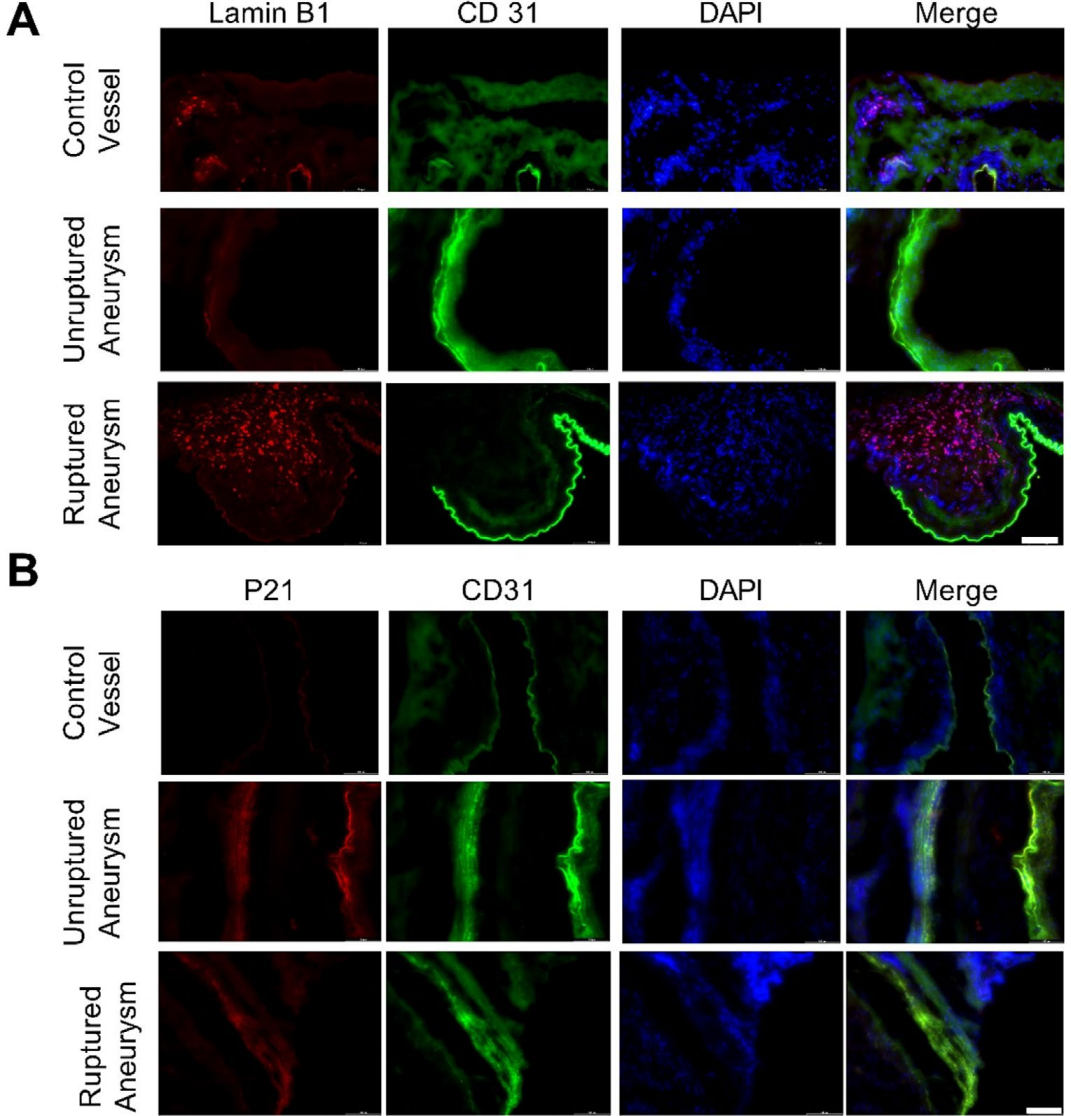
Immunofluorescence staining for (A) aging marker Lamin B1 and (B) senescence marker P21 in control vessels and unruptured and ruptured aneurysm tissue. Scale bar = 100 μm.

The immunofluorescence staining seemed to show enhanced Lamin B1 expression in aneurysm tissue compared to the control vessel (Figure 2A). In control vessels, CD31‐positive cells showed Lamin B1 expression (Figure 2A). In unruptured aneurysm tissue, a few CD31‐positive cells also presented Lamin B1 expression; however, Lamin B1 expression was absent in CD31‐positive cells in ruptured aneurysm (Figure 2A). Enhanced Lamin B1 expression was found to be colocalized with CD1b‐positive cells (data not shown). Immunofluorescence staining seemed to present enhanced expression of P21 in aneurysm tissue compared to control vessels.

**FIGURE 3:**
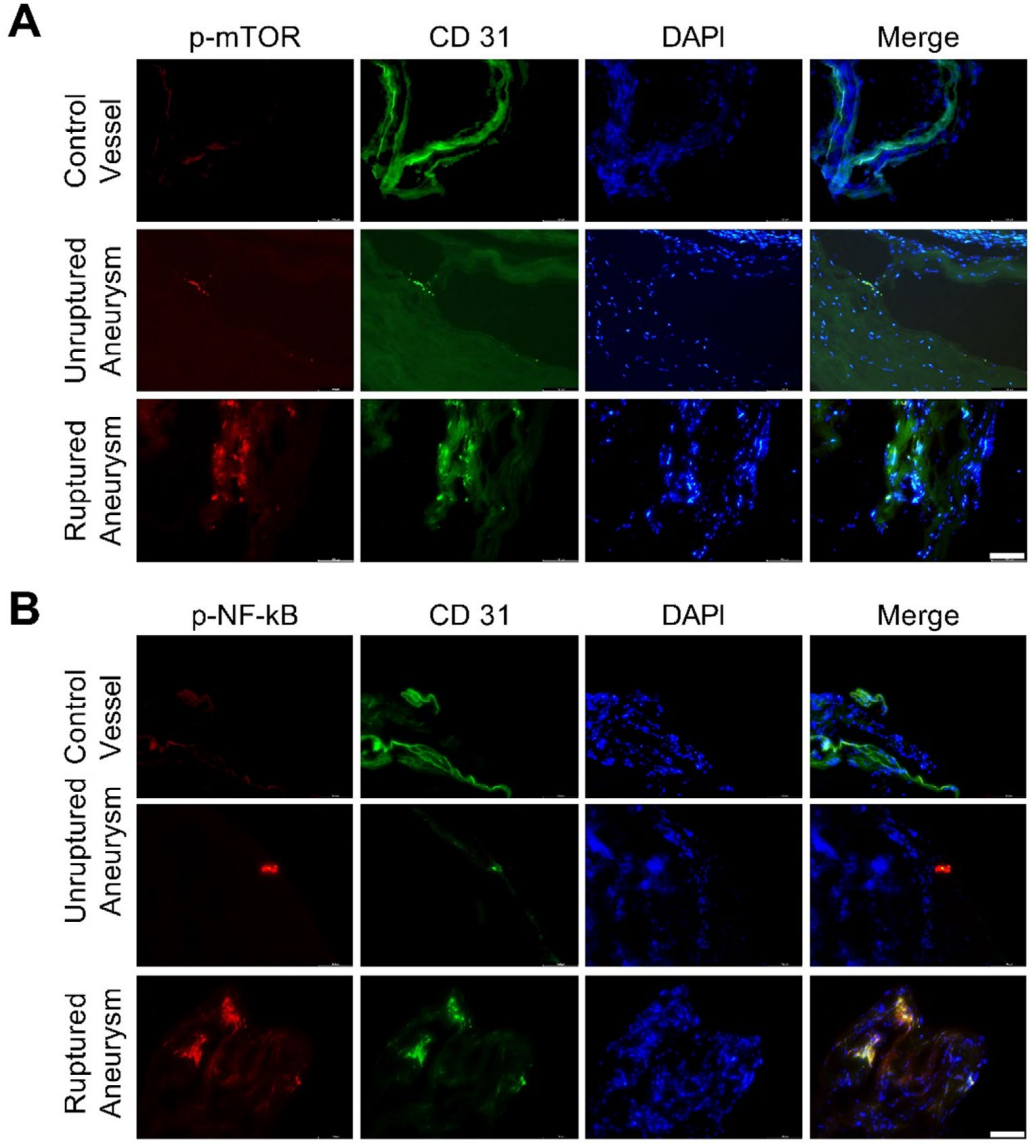
Immunofluorescence staining for (A) p‐mTOR and (B) p‐NF‐κB in the control vessel and unruptured and ruptured aneurysms. Scale bar = 100 μm.

The mTOR and NF‐κB pathways are known to contribute to aging, senescence, and inflammation (Garcia‐Garcia et al. 2021; Mannick and Lamming 2023). The expression of p‐mTOR and p‐NF‐κB looked higher in aneurysm tissue compared to the non‐diseased control vessel (Figure 3).

**FIGURE 4:**
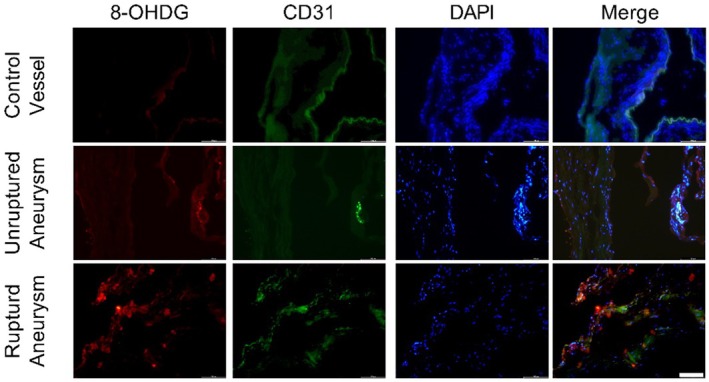
Immunofluorescence staining for oxidative stress‐induced DNA damage marker 8‐OHDG. Scale bar = 100 μm.

Cardiovascular risk factors such as smoking, alcohol abuse, and obesity can induce oxidative stress, which results in oxidative stress‐induced DNA damage, leading to telomere attrition and senescence (Li et al. 2022; Astuti et al. 2017; Clemente et al. 2019; Coluzzi et al. 2014). Therefore, immunofluorescence staining for the oxidative stress‐induced DNA damage marker 8‐OHDG was performed. Oxidative stress‐induced DNA damage seemed higher in aneurysm tissue compared to non‐diseased control vessels (Figure 4).

**FIGURE 5:**
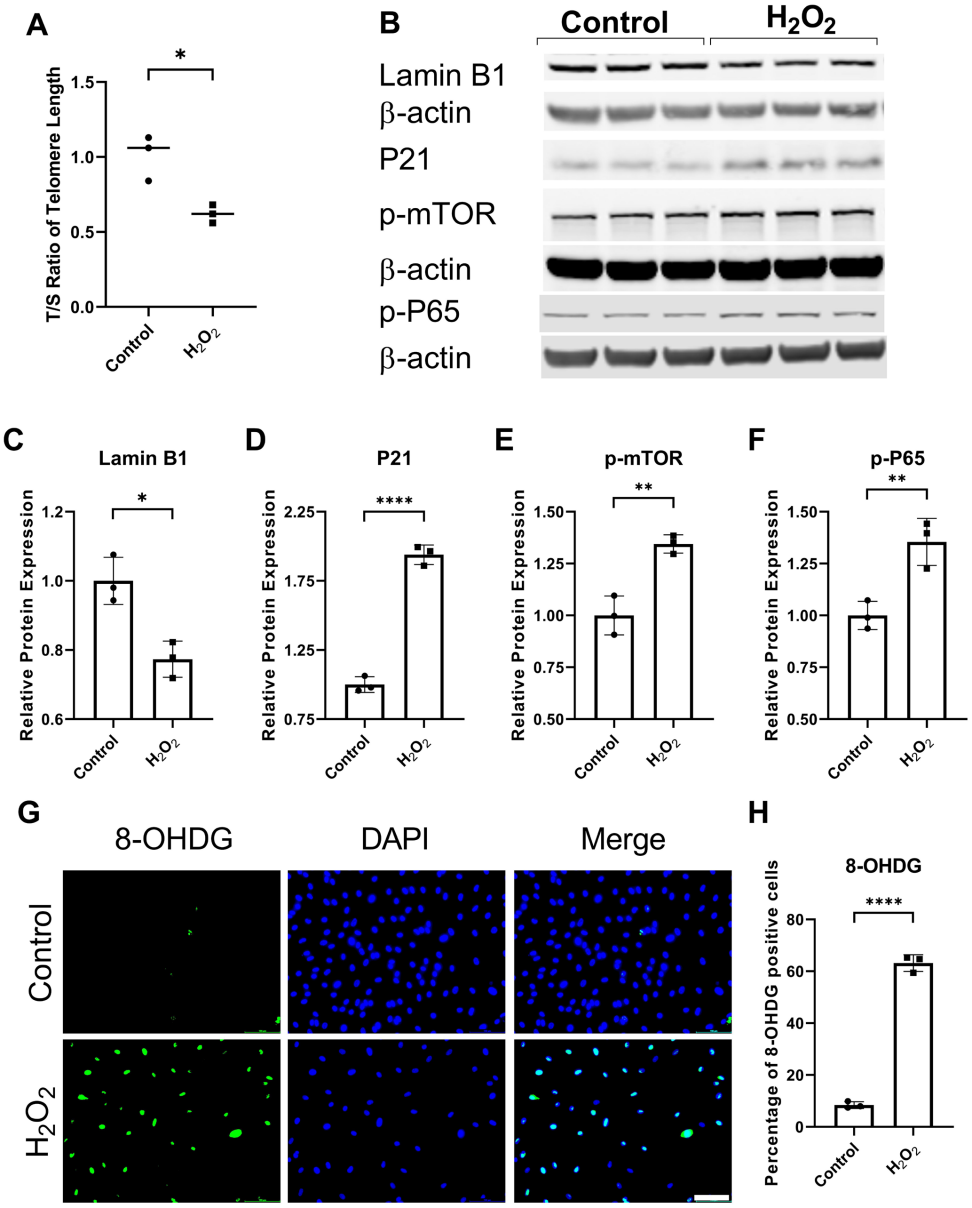
Oxidative stress induced accelerated molecular aging in endothelial cells. (A) Telomere attrition in endothelial cells exposed to H_2_O_2_. (B) Western blot showing protein expression of Lamin B1, P21, p‐mTOR, and p‐NF‐κB. Quantification of relative protein expression of (C) P21, (D) Lamin B1, (E) p‐mTOR, and (F) NF‐κB. (G) Immunofluorescent staining for 8‐OHDG in endothelial cells treated with oxidative stress. (H) Quantification of 8‐OXO positive endothelial cells. Scale bar = 100 μm. *n* = 3, *p < 0.05, **p < 0.01, ****p < 0.0001.

To study the effect of oxidative stress, endothelial cells were treated with 200 μM H_2_O_2_. H_2_O_2_ treatment reduced telomere length in endothelial cells (T/S ratio: Control = 1.01 ± 0.15, H_2_O_2_ = 0.62 ± 0.06, *n* = 3, *p < 0.05, Figure 5A). The expression of aging marker Lamin B1 was significantly decreased (Table 2; Figure 5B,C), and the expression of senescence marker P21 was significantly elevated (Table 2; Figure 5B,D) in endothelial cells exposed to H_2_O_2_. Oxidative stress also upregulated p‐mTOR (Table 2; Figure 5B,E) and p‐NF‐κB (Table 2; Figure 5B,F) expression in endothelial cells. Immunofluorescence staining showed significantly increased 8‐OHDG expression in H_2_O_2_‐treated endothelial cells (Percentage of 8‐OHDG positive cells: Control = 8.41 ± 1.30, H_2_O_2_ = 63.15 ± 3.21, *n* = 3, ****p < 0.0001, Figure 5G,H).

**TABLE 2:** Relative protein expression of Lamin B1, P21, p‐mTOR, and p‐NF‐κB (*n* = 3).

Name	Control (mean ± SD)	H_2_O_2_ (mean ± SD)	*p*
Lamin B1	1.00 ± 0.07	0.77 ± 0.05	< 0.05
P21	1.00 ± 0.06	1.94 ± 0.07	< 0.0001
p‐mTOR	1.00 ± 0.09	1.34 ± 0.04	< 0.01
p‐NF‐κB	1.00 ± 0.07	1.36 ± 0.11	< 0.01

## 2 Discussion

Aging initiates various cellular and molecular mechanisms that contribute to cardiovascular diseases (Ajoolabady et al. 2023). Vascular aging starts as early as childhood and continues throughout life. In the current explorative study, we investigated molecular aging and activation of signaling pathways, namely mTOR and NF‐κB associated with aging and inflammation, in unruptured and ruptured aneurysm tissue compared to non‐diseased control vessels. We report accelerated molecular aging, increased oxidative stress‐induced DNA damage, and the activation of mTOR and NF‐κB pathways in aneurysmal vascular tissue.

To determine molecular aging, we quantified telomere length and analyzed the expression of aging‐associated marker Lamin B1 in non‐diseased control vessels and aneurysmal vascular tissue. Both telomere quantification and Lamin B1 expression showed accelerated molecular aging in aneurysm tissue. The risk factors for intracranial aneurysm formation and rupture, such as smoking, alcohol abuse, hyperglycemia, and hypercholesterolemia, have been associated with telomere shortening (Turner et al. 2019; Astuti et al. 2017). Shortened telomere length and rate of telomere attrition have been associated with cardiovascular diseases such as hypertension, atherosclerosis, vascular dementia, and coronary heart disease (Turner et al. 2019). Previous studies in atherosclerosis and coronary artery disease have shown telomere attrition of approximately 6%–10% in affected tissue and leukocytes compared to healthy controls (Willeit et al. 2010; Nzietchueng et al. 2011). In our study, we observed a telomere reduction of approximately 20% in unruptured and 26% in ruptured intracranial aneurysm tissue compared to non‐diseased vessels. These findings suggest that aneurysmal tissue exhibits a degree of molecular aging greater than that reported in other chronic vascular diseases. While these conditions share common drivers of telomere attrition, including oxidative stress, inflammation, and endothelial dysfunction, the additional influence of anatomical location and localized pulsatile high hemodynamic stress in aneurysmal walls may further contribute to telomere decrease. Telomere shortening and DNA damage can trigger senescence in endothelial cells (Ungvari et al. 2013). Immunofluorescence staining revealed enhanced expression of the senescence marker P21 in aneurysm tissue (Figure 2B,C). It has been suggested that senescence induction is a key mechanism through which DNA damage contributes to vascular aging (Uryga and Bennett 2016). Senescent cells halt cell division but remain metabolically active and exhibit a senescence‐associated secretory phenotype characterized by increased expression and release of MMPs, growth factors, and pro‐inflammatory molecules such as IL‐1β, IL‐6, TNF‐α, and MCP‐1. Some of these SASP factors are known to contribute to aneurysm pathogenesis and rupture. Experimental animal studies have shown that intracranial aneurysm formation, progression, and rupture could be mitigated by inhibiting SASP factors such as IL‐1β, TNF‐α, MCP‐1, and MMPs (Moriwaki et al. 2006; Starke et al. 2014; Aoki et al. 2007, 2009). While our analysis focused on tissue‐level changes, the potential diagnostic utility of circulating SASP factors or telomere length in peripheral blood should be further investigated. If validated, these markers could offer non‐invasive tools to assess aneurysm rupture risk and guide clinical management.

Oxidative stress induces DNA damage (Figure 5G,H) (Zhou, Khan, et al. 2023), resulting in telomere shortening (Figure 5A) and premature stress‐induced senescence (Figure 5B–D) (Zhou, Khan, et al. 2023; Coluzzi et al. 2014). Immunofluorescence revealed enhanced oxidative stress‐induced DNA damage in aneurysm tissue compared to non‐diseased control vessels (Figure 4). Interestingly, the DNA repair pathway in endothelial cells has been reported to be less efficient (Ungvari et al. 2013). Oxidative stress and oxidative stress‐induced DNA damage can activate mTOR and NF‐κB pathways (Zhou, Khan, et al. 2023). The aneurysm tissue showed increased expression of p‐mTOR and p‐NF‐κB (Figure 3). The activation of NF‐κB and mTOR pathways has been implicated in aging and senescence (Garcia‐Garcia et al. 2021; Mannick and Lamming 2023; Tichy et al. 2021). Various animal models have confirmed the contribution of the mTOR pathway to aging (Mannick and Lamming 2023). Blocking mTOR and its downstream signaling molecule S6 increased animals' life and health span (Mannick and Lamming 2023). Additionally, mTOR activation blocks autophagy, resulting in the accumulation of misfolded proteins, which can consequently increase oxidative stress. It is worth noting that pharmacological interventions that promote autophagy reverse arterial aging (LaRocca et al. 2013). By inhibiting mTOR and blocking mTOR translation using mTOR‐siRNA attenuated atherosclerotic plaque rupture in vivo (Zhai et al. 2014). mTOR pathway is also known to contribute to inflammation (Weichhart et al. 2015). mTOR pathway is involved in the polarization of immune cells (Weichhart et al. 2015). mTOR inhibition promotes anti‐inflammatory and pro‐reparative phenotypes in microglia and monocytes. Furthermore, blocking mTOR favors T‐regs differentiation. Moreover, studies have suggested cross‐talk between mTOR and NF‐κB pathways (Dan et al. 2008; Xu et al. 2021). Dan et al. (2008) demonstrated that mTOR, acting downstream of Akt, regulates NF‐κB activity through direct stimulation of IKK in PTEN‐deficient cancer cells, suggesting a mechanistic link involving mTOR‐IKK interaction and Ratpor dependance. mTOR inhibitors Rapamycin and Rapalink‐1 have been reported to suppress NF‐κB activation (Dan et al. 2008; Zhou, Li, et al. 2023). NF‐κB is a transcription factor for inflammatory factors. NF‐κB plays an important role in intracranial aneurysm formation and rupture (Khan et al. 2023). Furthermore, NF‐κB contributes to accelerated aging (Garcia‐Garcia et al. 2021). The activation of NF‐κB has been reported to reduce telomere length and cause senescence in different cell models (Tichy et al. 2021). In addition, NF‐κB activation promotes the expression of senescence‐associated proteins, including P21 (Nicolae et al. 2018). Blocking NF‐κB attenuated telomere attrition, reduced cellular senescence, and improved lifespan in animal models (Garcia‐Garcia et al. 2021; Tichy et al. 2021; Tilstra et al. 2012; Zhang et al. 2021).

Because enhanced oxidative stress‐induced DNA damage was observed in aneurysm vessels than in non‐diseased control vessels (Figure 4), we exposed endothelial cells to H_2_O_2_ to investigate whether oxidative stress can lead to telomere attrition. Our in vitro experiments showed that H_2_O_2_ treatment accelerated molecular aging (Figure 5A–D) and increased the expression of activated NF‐κB and mTOR in endothelial cells (Figure 5B,E,F). Previous studies have shown that cardiovascular risk factors such as smoking, alcohol abuse, and obesity promoted accelerated aging and induced senescence in endothelial cells (Turner et al. 2019; Khan et al. 2024; Li et al. 2022). Moreover, it has been reported that the generation of O^2−^ from mitochondria increases in aged arteries, resulting in increased oxidative stress, which has been shown to promote inflammation in the arteries of aged rats by activating NF‐κB in endothelial cells (Ungvari et al. 2007).

Aging begins as early as childhood, with the gradual shortening of telomere occurring naturally during each cell division (Turner et al. 2019). However, in vascular tissue, this process may be accelerated by external factors such as oxidative stress, inflammation, and cardiovascular risk exposures (Turner et al. 2019; Li et al. 2022; Astuti et al. 2017; Clemente et al. 2019). Whether telomere shortening is a primary cause of aneurysm formation or a consequence of chronic vascular stress remains a subject of debate. Evidence suggests a bidirectional relationship: oxidative and inflammatory insults can hasten telomere attrition (Turner et al. 2019; Liu et al. 2019; Coluzzi et al. 2014), while critically shortened telomeres themselves can trigger endothelial dysfunction, cellular senescence, and inflammation (Bhayadia et al. 2016; Lex et al. 2020). This creates a self‐perpetuating cycle that can contribute to aneurysm pathogenesis. Understanding this dynamic is essential, as it positions telomere biology as both a diagnostic marker and a potential therapeutic target.

Despite the strength of our findings, the study has some limitations. First, while 8‐OHDG staining confirmed oxidative stress‐induced DNA damage, we did not directly measure ROS levels due to tissue preservation constraints. However, 8‐OHDG is an established and widely used surrogate marker for oxidative stress‐induced DNA damage. Future studies using ROS sensitive probes in fresh or live tissue models may further strengthen these observations. Also, it should be noted that hydrogen peroxide is not the most natural oxidizing agent, therefore, the in vitro data should be carefully interpreted. Second, our data do not distinguish between telomere attrition in endothelial cells and smooth muscle cells. Single‐cell RNA sequencing or lineage tracing techniques could address this in future investigations. Third, arteria meningea media served as a practical control, differences in embryonic origin and flow dynamics may influence results and should be considered when interpreting vascular aging comparisons. Fourth, detailed information on comorbidities such as smoking and hypertension was not available for all control samples, as these tissues were collected during unrelated surgical procedures. Finally, while we identified SAP‐related senescence markers in aneurysmal tissue, the in vivo secretion of specific factors could not be directly measured. Nonetheless, our findings align with previous studies linking SASP activity in aneurysm progression.

## 3 Conclusion

The objective of the current study was to investigate molecular aging in aneurysm tissue. Molecular aging was accelerated in vascular tissue of unruptured and ruptured aneurysms than in non‐diseased control vessels. Oxidative stress‐induced DNA damage was increased in aneurysm vessels. Aneurysm tissue also showed increased expression of aging and inflammation‐associated pathways p‐mTOR and p‐NF‐κB. Oxidative stress reduced the length of telomeres, lowered the aging marker Lamin B1, and increased P21 expression in vitro endothelial cell models, similar to human aneurysm tissue.

## 4 Method

To investigate accelerated aging, oxidative stress‐induced DNA damage, and the activation of signaling pathways in intracranial aneurysms, we harvested unruptured and ruptured aneurysm tissue during surgery in patients undergoing treatment for brain aneurysms (Muhammad and Niemelä 2019). The control tissue was harvested from arteria meningea media; a normal vessel usually sacrificed during the surgical approach.

### 4.1 Telomere Length Quantification

Endothelial cells were treated with 200 μM H_2_O_2_. Cells cultured with only endothelial cell medium were used as controls. The medium was changed every second day. DNA was extracted from endothelial cells after 4 days of treatment and from non‐diseased control vessels and unruptured and ruptured aneurysm tissue using the innuPREP DNA Mini Kit (845‐KS‐1042050; Analytik Jena, Jena, Germany). One nanogram of DNA was used for telomere length measurements. Primers used for the qPCR are provided in Table S1. AceQ SYBR qPCR Master Mix (Q111‐03; Vayzme, Nanjing, China) was used for the qPCR. The qPCR protocol was an initial denaturation at 95°C for 10 min, followed by 40 cycles of 95°C for 15 s and 60°C for 1 min. The qPCR protocol was concluded with a melting curve. The relative telomere length for endothelial cells exposed to H_2_O_2_ was calculated as the telomere to single‐copy gene (IFNB1) ratio (T/S ratio). The telomere length for human tissue samples was quantified using a telomere quantification kit (SC‐8918; Provitro AG, Berlin, Germany). The comparative ΔCT method was used to quantify telomere length.

To ensure consistency across methodologies, a cross‐validation on patient samples using both approaches was performed, and similar group‐wise trends in telomere length were observed. Therefore, we report results in KBs (for clarity and standardization), and in vitro results in T/S ratio (due to differences in sample type and assay suitability).

### 4.2 Immunofluorescence (IF) Staining

Fresh tissue was cut on a cryotome and directly frozen at −80°C until use. For IF staining, the tissue was fixed by incubation with 4% ice‐cold PFA for 10 min. Then, it was permeabilized with 0.3% Triton X‐100 in TBS on ice for 10 min. After three rounds of washing with 0.1% Triton X‐100 for 5 min each, the tissue was incubated for 1 h in 5% BSA in TBS for blocking. Afterward, the tissue was washed again as before and then incubated in the primary antibody (Table S2) in 0.1% BSA and 0.1% Triton X‐100 in TBS at 4°C overnight. On the next day, the tissue was washed again as before and then incubated in the secondary antibody (Table S2) in 0.1% Triton X‐100 in TBS at room temperature for 1 h. Finally, the tissue was washed the last time as before and then fixed with ROTI Mount FluorCare (HP19.1; Carl Roth GmbH+Co. KG, Karlsruhe, Germany) and sealed.

Endothelial cells were seeded in a 96‐well plate (5000 cells/cm^2^). The following day, the medium was changed, and the cells were exposed to oxidative stress for 2 h. Then, they were washed thrice with PBS and fixed in 4% PFA for 15 min. The cells were permeabilized with 0.1% Triton X‐100 for 10 min. The cells were blocked with 5% BSA for 1 h at RT to prevent nonspecific antibody binding. After that, the cells were incubated with the primary antibody 8‐OHDG (1:500, Cat. No. BSS‐BS‐1278R; BIOSS, Woburn, MA, USA) in 5% BSA on a shaking platform overnight. The next day, after washing three times with PBS, the cells were incubated with a secondary antibody (1:1000, Alexa Fluor 488, Cat. No. ab150077) at RT for 60 min. SlowFade Gold Antifade Mountant with DAPI (62248; Thermo Fisher, Waltham, MA, USA) was used for nuclear staining. The images were captured at 20× magnification.

### 4.3 Western Blot

For protein analysis, endothelial cells were treated with H_2_O_2_. The cells treated with only endothelial cell medium were used as a control. After 24 h treatment, RIPA buffer was used to extract the total protein. The DC Protein Assay Kit (500‐0116; Bio‐Rad, Hercules, CA, USA) was used according to the manufacturer's instructions to determine protein concentrations calorimetrically on a Paradigm micro‐plate reader. The SDS‐PAGE was performed with 25 μg of total protein in reducing conditions on a 12% sodium dodecyl sulfate‐polyacrylamide gel. The running conditions were 60 V for 20 min followed by 110 V for 30–60 min. After that, the proteins were transferred onto a nitrocellulose membrane at 250 mA for 120 min. The membranes were blocked with 5% BSA in 0.05% TBST for 1 h. Following, the membranes were incubated with primary antibodies (Table S2) on a shaking platform overnight at 4°C. The next day, the membranes were washed thrice with TBST. Then, the membranes were incubated with secondary antibodies (Table S2) at RT for 1 h. Image J was used to calculate the densitometry. β‐actin was used as a housekeeping gene. The experiment was performed in triplicate.

### 4.4 Statistical Analysis

For statistical analysis, a *t*‐test was performed for comparing two groups, and for multiple groups, ANOVA followed by post hoc Tukey's test was performed. Data were first assessed for normality using the Shapiro–Wilk test. Since the telomere data did not follow a normal distribution and variance were unequal (confirmed by Bartlett's test, *p* < 0.05), we used the Kruskal‐Wallis test for multiple group comparison. Dunn's multiple comparison post hoc test was applied to assess pairwise differences. Significance was defined as *p* < 0.05.

## Author Contributions

Conceptualization: S.M. and D.K. Methodology: X.L. and M.H. Data collection and curation: D.K. and X.L. Formal analysis and investigation: X.L. and H.Z. Writing – original draft preparation: D.K. Writing – review and editing: S.M. Visualization: X.L. Supervision: S.M.

## Ethics Statement

Ethical approval (Studien‐Nr.: 2019‐787‐bio) was obtained for collecting and using liquid and solid biopsies.

## Consent

Informed consent was obtained from the patients. In cases where patients could not give informed consent, informed consent was obtained from their proxies.

## Conflicts of Interest

The authors declare no conflicts of interest.

## Supporting information


**Table S1:** Primer list.
**Table S2:** Primary and secondary antibodies.

## Data Availability

The data that support the findings of this study are available on request from the corresponding author. The data are not publicly available due to privacy or ethical restrictions.
