# Supplementary material for: First Direct Evidence of Accelerated Molecular Aging in Intracranial Aneurysmal Tissue
Source: Aging Cell. 2025 Sep 30;24(11):e70231. doi: 10.1111/acel.70231 (PMC12611279; doi:10.1111/acel.70231)
Supplement: Supplementary file 1 — Table S1: Primer list. Table S2: Primary and secondary antibodies. [file ACEL-24-e70231-s001.docx]

Table S1. Primer list

| Target gene | Sense 5’ -3’ | Antisense 5’ -3’ |
| --- | --- | --- |
| TEL | CGGTTTGTTTGGGTTTGGGTTTGGGTTTGGGTTTGGGTT | GGCTTGCCTTACCCTTACCCTTACCCTTACCCTTACCCT |
| IFNB1 | GGTTACCTCCGAAACTGAAGA | CCTTTCATATGCAGTACATTAGCC |
|  |  |  |

Table S2. Primary and secondary antibodies

| Antibody | MW (kDa) | Brand | Catalog number | Concentration (WB) | Concentration (IF) |
| --- | --- | --- | --- | --- | --- |
| Lamin B1 | 66 | Abcam | Ab16048 | 1:1000 | 1:200 |
| CD 31 | 130 | Cell Signaling | 3528S |  | 1:250 |
| P21 | 21 | Cell Signaling | 2947S |  | 1:200 |
| p-mTOR | 289 | Cell Signaling | 2971S | 1:1000 | 1:200 |
| p-NF-κB | 65 | Cell Signaling | 3033S | 1:1000 | 1:200 |
| 8-OHDG |  | BIOSS | BSS-BS-1278R | 1:500 | 1:200 |
| β-actin | 45 | Cell Signaling | 4970S | 1:1000 |  |
| IRDye 680RD |  | LiCor | 926-68072 | 1:10,000 |  |
| IRDye 800CW |  | LiCor | 926-32211 | 1:10,000 |  |
| Alexa Fluor 488 |  | Cell Signaling | 4408 |  | 1:1000 |
| Alexa Fluor 594 |  | Cell Signaling | 8889 |  | 1:1000 |
